# Left Atrial Anterior Wall Scar-Related Atrial Tachycardia in Patients after Catheter Ablation or Cardiac Surgery: Electrophysiological Characteristics and Ablation Strategy

**DOI:** 10.3390/jcdd9080249

**Published:** 2022-08-05

**Authors:** Hao Wang, Siqi Xi, Jindong Chen, Tian Gan, Weiye Huang, Ben He, Liang Zhao

**Affiliations:** 1 Department of Cardiology, Shanghai Chest Hospital, Shanghai Jiao Tong University, Shanghai 200003, China; 2 Cardiac Electrophysiology Department, Boston Scientific Company, Shanghai 200023, China

**Keywords:** atrial tachycardia, left atrial anterior wall, iatrogenic intervention, ablation, high-density mapping

## Abstract

**Background:** The mechanisms of atrial tachycardia (AT) related to the left atrial anterior wall (LAAW) are complex and can be challenging to map in patients after catheter ablation for atrial fibrillation (AF) or cardiac surgery. We aimed to investigate the electrophysiological characteristics AT and to devise an ablation strategy. **Methods and Results:** We identified 31 scar-related LAAW reentrant ATs in 22 patients after catheter ablation for AF or cardiac surgery. Activation maps of the left atrium (LA) or both atria were obtained using a high-density mapping system, and the precise mechanism and critical area for each AT were analyzed. Patients were followed up regularly in a clinic. After analyzing the activation and propagation of each AT, the scar-related LAAW ATs were classified into three types, based on mechanisms related to: (1) LAAW conduction gap(s) in 19 LA macro-reentrant ATs; (2) LAAW epicardial connection(s) in 11 LA or bi-atrial ATs; and (3) LAAW local micro-reentry in 1 LAAW AT. Multiple ATs were identified in seven patients. Effective ablation (termination or circuit change of AT) was obtained in 30 ATs by targeting the critical area identified by the mapping system. During 16.0 ± 7.6 months follow-up, recurrent AT occurred in two patients. **Conclusions:** Three mechanisms of scar-related AT of LAAW were identified, most of which were related to LAAW conduction gaps. Notably, epicardial AT or bi-atrial AT comprised a nonnegligible proportion. A high-density mapping system could make it possible to determine the accurate mechanism of AT and serve as a guide following ablation.

## 1. Introduction

Left atrial anterior wall (LAAW) scars and lesions are common among patients after iatrogenic interventions such as catheter ablation for non-paroxysmal atrial fibrillation (AF) or cardiac surgery [1,2,3]. For example, LAAW linear ablation and complex fractionated atrial electrogram (CFAE) ablation is a frequently-used approach for non-paroxysmal AF [4], while left atrial (LA) septal and septal-roof incisions are common approaches in the left heart in cardiac surgery [5]. Iatrogenic scars and lesions on LAAW provide a potential substrate for atrial tachycardia (AT). The mechanism of scar-related ATs is usually complicated due to the non-uniformity of scars and the versatility of activation [6,7]. With the guidance of a high-density mapping system, we investigated the electrophysiological characteristics and ablation strategy of scar-related LAAW reentrant AT in patients after LAAW.

## 2. Materials and Methods

### 2.1. Study Population

From October 2019 to November 2021, the electrophysiological study (EPS) files of a consecutive cohort of patients undergoing catheter ablation for AT using the Rhythmia high-density mapping system (Boston Scientific, Marlborough, MA, USA) were reviewed. All patients with LAAW-related AT and a history of LAAW intervention were enrolled and analyzed. Written consent was obtained from every patient, and the study was approved by the Shanghai Chest Hospital Ethics Committee. The study was conducted in accordance with the Declaration of Helsinki (as revised in 2013).

### 2.2. Electrophysiological Study and Ablation Procedure

Before the procedure, anti-arrhythmic drugs were stopped for at least five half-lives. Transesophageal echocardiography was performed in each patient to exclude left atrial thrombus. Briefly, a 6F decapolar catheter was placed in the coronary sinus (CS) via the femoral vein under local anesthesia as a stable reference to local activation time. If the patient was in sinus rhythm before mapping, AT was induced by programmed CS pacing.

Electroanatomic mapping was conducted with an Orion multipolar catheter and the Rhythmia mapping system. (Boston Scientific, MA) If the atrial wave was preceding in proximal CS leads, right atrial (RA) mapping was conducted, whereas if the atrial wave was preceding in distal CS leads or RA mapping suggested LA-originated AT, the Orion catheter was advanced into LA via the transseptal approach. During mapping, an activation map was created under standard automatic beat acceptance criteria as follows: (1) cycle length (CL) variation less than 10 ms; (2) activation time difference between two CS electrograms within 5 ms; (3) catheter motion within 1 mm during the cardiac cycle; and (4) the distance between anatomical shell and electrode within 2 mm. The circuit of each AT was then analyzed to identify a detailed mechanism.

Pre-existing linear lesions were identified by conduction block of lines shown as sharp changes in activation time and color on the activation map. The scar was identified by voltage mapping. An endocardium with a voltage less than 0.1 mV was considered a scar, which was depicted in red on the activation map. A critical conduction gap was defined as a slow conduction area formed by conduction barriers on both sides, which was demonstrated as a decrease in the activation area within the AT cycle, as illustrated by Lumipoint^TM^, and further identified by the Skyline tool (Boston Scientific, MA). Bi-atrial tachycardia (biAT) was diagnosed if the circuit used both LA and RA through two interatrial connections [8]. Epicardial AT was diagnosed if the wavefront propagation of the reentrant circuit showed a ‘jump-frog’ pattern with focal activation after bypassing the atrial conduction barrier. The following characteristics may be helpful in recognizing epicardial conduction connection-related AT: (1) a missing TCL of >10%; (2) a local breakthrough site >10 mm remote from a collision site; (3) the possibility of bi-atrial AT should be considered when local breakthrough was observed near inter-atrial connections including the Bachmann’s bundle, fossa ovalis, posteroinferior interatrial connection, and coronary sinus ostium; (4) the earliest local breakthrough on endocardium activated in an areal pattern simultaneously rather than in a point-like pattern, as revealed by a high-density mapping system; and (5) effective ablation should be performed in an areal pattern covering the endocardial local breakthrough site. In our study, all 11 Type 2 ATs conformed to the characteristics mentioned above, and effective ablation was achieved in 10 ATs. Micro-reentrant AT, as a subtype of focal AT, was defined as reentrant atrial activation with a circuit <2 cm in diameterspreading centrifugally [9]. Scar-related LAAW AT was diagnosed if: (1) the LAAW scar identified via mapping was consistent with previous LAAW intervention history; and (2) the mechanism of AT was related to the LAAW scar.

After the mechanism of AT was confirmed, for macro-reentrant AT, linear ablation was performed across the critical area, which was the narrowest part of the circuit with slow conduction. For micro-reentrant AT, ablation was performed targeting the most fractionated electrogram along the reentrant path or in a linear fashion from the site of the circuit to an anatomical barrier or an area of conduction block. An irrigated catheter (Boston Scientific, MA) was used for ablation (43 °C, 35 W, irrigation rate at 12 mL/min). Heparin (100 IU/kg) was administrated to maintain an active clotting time of between 300 and 350 s. Effective ablation referred to the cessation of AT and restoration of sinus rhythm or change of AT circuit. According to our own definition, circuit change includes changes in the conduction path and sequence and can be further divided into: (1) a change in the local conduction connection or exit without altering the global propagation of AT; (2) global propagation alteration; and (3) reversal in the conduction sequence without changing the circuit path. During the ablation procedure, circuit changes can manifest as: (1) changes in the AT cycle length; (2) changes in the activation sequence of CS electrodes; and (3) changes in the AT mechanism, as revealed by high-density mapping.

The endpoint of the ablation procedure included: (1) the termination of AT and restoration of sinus rhythm; (2) subsequent substrate modification targeting bystander conduction gaps, the slow conduction zone, or a local area with complex fractionated atrial electrograms, as revealed by the Skyline^TM^ and Lumipoint^TM^ tools in the Rhythmia mapping system, which is a potential substrate facilitating other ATs; and (3) confirmation of a bi-directional block of the LAAW line.

After sinus rhythm was restored, bidirectional conduction block of the LAAW line was confirmed when the following criteria were met. (1) Double potentials could be observed along the LAAW line under sinus rhythm; (2) when pacing close to the ablation line, an abrupt change of activation time of at least 100 ms and reversal in the direction of activation on the opposite side of the LAAW line could be observed; and (3) activation propagation of the left atrium under sinus rhythm confirmed via a high-density mapping system showing a complete block of LAAW line without local activation breakthrough across the LAAW line.

### 2.3. Follow Up

During hospitalization, electrocardiograph (ECG) monitoring was applied to all patients after the procedure. After discharge, patients underwent regular clinic visits and were assessed with 12-lead ECG and 24 h Holter monitoring after the procedure.

### 2.4. Statistic Analysis

Normally distributed continuous variables were expressed as mean ± standard deviation and abnormally distributed continuous variables as medians (range). Comparisons of continuous variables were conducted by Students’ *t*-test or Mann-Whitney U test. A *p* < 0.05 was considered statistically significant. Statistical analyses were performed by SPSS 26.0 (IBM Corp., Armonk, NY, USA).

## 3. Results

### 3.1. Study Population and LAAW Intervention

Between October 2019 to November 2021, this study enrolled 22 patients who underwent RFCA for AT after LAAW intervention. LAAW intervention included RFCA for persistent AF in 13 patients and cardiac surgery in the other 9 patients. All patients after cardiac surgery underwent mitral valve replacement (MVR); additional procedures included aortic valve replacement (AVR) in two patients, tricuspid valve plasty (TVP) in three patients, and TVP plus MAZE IV in one patient. The mean left atrial diameter was 44.9 ± 4.57 mm and the mean left ventricular ejection fraction was 59.5 ± 9.3%. Eighteen patients had persistent AT, while the other four patients had paroxysmal AT and were induced by CS pacing during the electrophysiological study. The mean duration between LAAW intervention and AT ablation was 24.7 ± 6.9 months after RFCA for AF and 31.3 ± 9.8 months after cardiac surgery. (*p* = 0.076). The baseline characteristics of the patients are summarized in Table 1.

### 3.2. AT Mapping Results

In total, 31 LAAW-related ATs were identified in 22 patients, and multiple mechanisms were identified in 7 patients. The index procedure was for LAAW-related ATs in 21 patients, while in one patient, the index procedure was for typical atrial flutter, which converted to LAAW-related AT after successful ablation of typical atrial flutter. In all 31 LAAW-related ATs, LA + RA mapping was performed in 14 ATs in 26.2 ± 4.6 min, and LA mapping alone was performed in the other 17 ATs in 17.3 ± 4.5 min. The mean numbers of mapping points were 10,313.9 ± 2436.6 in LA and 6858.9 ± 1368.7 in RA. The mean total cycle length (TCL) was 262.4 ± 61.0 ms, and manual annotation was performed in only 23 points in three AT maps. The results of mapping and ablation are summarized in Table 2.

### 3.3. LAAW Linear Lesions and Scars

Linear lesions and/or scar areas on LAAW endocardium associated with the previous iatrogenic intervention were observed in all 22 patients during mapping. The patterns of linear lesions included: (1) from the right superior pulmonary vein (RSPV) to the anterolateral mitral valve annulus (MVA) in 17 patients; (2) from the LA septum extending to the LA roof, consistent with LA septal-roof incision in four patients after cardiac surgery; and (3) a minor linear lesion near MVA in one patient after cardiac surgery; (4) additional linear lesions were identified in four patients, including the left superior pulmonary vein (LSPV)-MVA line in two patients and the LA roof line from RSPV to LSPV in two patients. The mean length of all 26 lesion lines was 55.1 ± 12.8 mm. In nine patients after cardiac surgery, superior transseptal approach was performed in four patients (44.4%), and transseptal approach was performed in the other five patients (55.6%). Twelve ATs were identified in these nine patients, including four Type 1.1 ATs, two Type 1.3 ATs, four Type 2.1 ATs, one Type 2.2 AT, and one Type 3 AT.

LAAW scars consistent with surgical procedures were identified in two patients after cardiac surgery, including one with a LA septal-roof linear lesion and one with a minor LAAW linear lesion. The areas of LAAW scars were 342 mm^2^ and 315 mm^2^, respectively. The mechanisms of ATs in these two patients were not scar-related.

### 3.4. Conduction Gaps and the Patterns of AT Activation Circuits

In total, 24 conduction gaps were identified from 20 ATs. Multiple conduction gaps were observed in three patients, of which two conduction gaps were identified in two patients and three conduction gaps in the other patient. The conduction gaps were located (1) at the MVA end of the LAAW line (11 ATs); (2) at the RSPV end of the LAAW line (7 ATs); (3) in the middle of the LAAW line (2 ATs); (4) in the middle of LSPV-MVA line (4 ATs); and (5) between septal and LAAW lesion lines caused by surgical incision (1 ATs).

**Figure 1:**
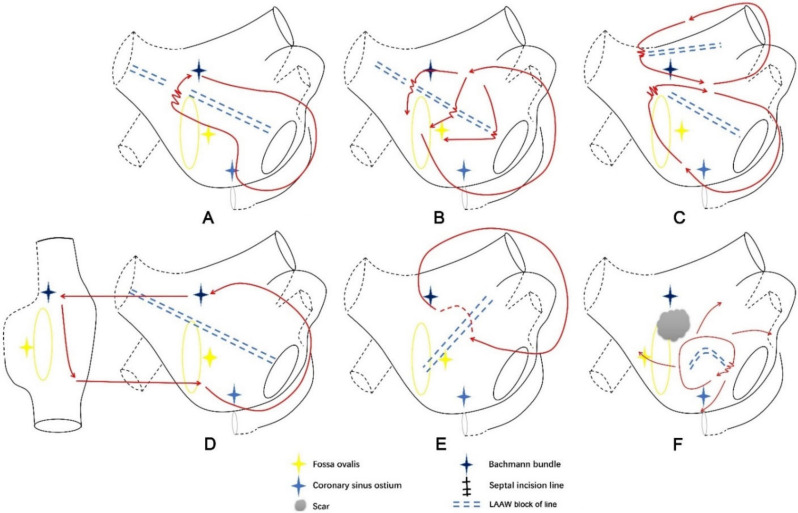
Simplified schemas of six subtypes of the left atrial anterior wall (LAAW) atrial tachycardia (AT) reentrant circuits. (**A**), Type 1.1: Single-looped macro-reentrant AT with one single LAAW gap as the critical area. The propagation of AT ran across the LAAW gap and around the mitral valve annulus (MVA). (**B**), Type 1.2: The propagation course was similar to Type 1.1, except that there were multiple LAAW gaps. The propagation of AT ran around the MVA and went across all the LAAW gaps. (**C**), Type 1.3: Dual-looped AT with LAAW conduction gap(s) as the critical area. The wavefront simultaneously propagated around a pre-existing LA roof scar and ran around the LAAW scar and MVA. (**D**), Type 2.1: Single-looped bi-atrial reentrant ATs whose circuit used both LA and RA. The wavefront activated RA at the superior septum consistent with RA insertion of BB, running down the septum and jumped to LA through fossa ovalis or coronary sinus ostium. Then the wavefront ran around the MVA counterclockwise to LAAW, returning to the LA insertion of BB to complete the circuit. (**E**), Type 2.2: LAAW epicardial macro-reentrant AT featured by an epicardial connection crossing the pre-existing linear lesion. The wavefront began at the LAAW breakthrough above the lesion line, ran around LA roof then down along LA posterior wall, and then went around LA lateral wall and across the lesion line via the epicardial connection. (**F**), Type 3: LAAW micro-reentrant AT. The circuit ran around a minor LAAW block of line near the MVA, and the wavefront of AT propagated in a centrifugal pattern. The red line with arrows indicated the circuit of AT; the jagged line indicated the wavefront passing the slow conduction area; the red dashed line indicated the epicardial bypass of the AT circuit.

The mechanisms of LAAW-related ATs were analyzed and categorized into three types as follows (Figure 1). Type 1: Left atrial macro-reentrant ATs with LAAW conduction gap(s) as the critical area. Nineteen ATs of this type were identified in 16 patients and further categorized into three subtypes.

**Figure 2:**
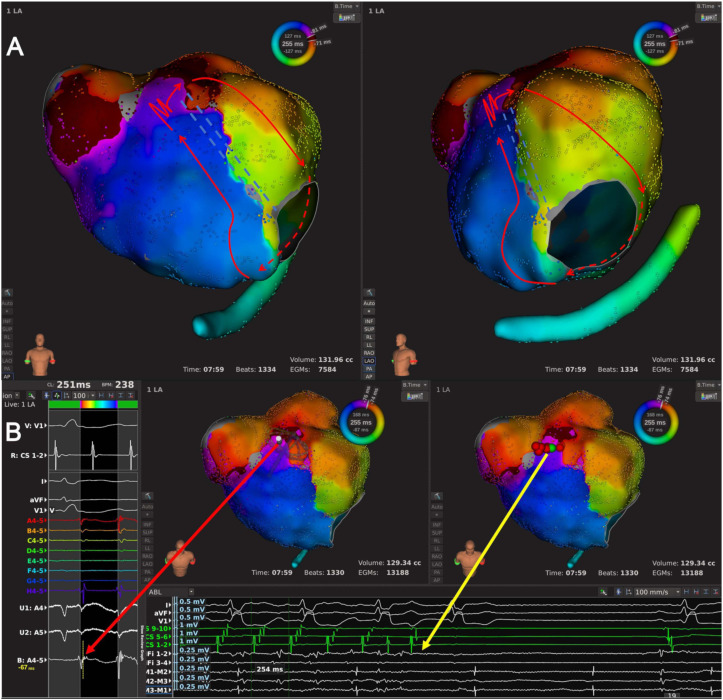
The high-density mapping of a Type 1.1 AT. (**A**) Activation map of a Type 1.1 AT from the anteroposterior view (left) and the left-anterior oblique view (right). The LA high-density mapping identified one LAAW block line stretching from the right superior pulmonary vein (RSPV) to the MVA, with a conduction gap near the RSPV end. The wavefront of AT ran along the LAAW line upwards in LA septum to jump across the LAAW gap, then ran toward MI and around the MVA clockwise, and returned to LA septum. The arrowed red lines indicated the AT circuit; the dashed red line indicated the AT circuit behind the atrial structure; the jagged red line indicated the wavefront passing the slow conduction area; the double-dashed blue line indicated the LAAW block of line. (**B**) Local map potentials of the LAAW gap during AT (left) and at the moment of AT termination (bottom). The red dots indicated the ablation points; the green dot indicated the termination site; the arrowed red and yellow lines indicated the local potentials during AT and at the moment of AT termination, respectively.

Type 1.1: This subtype of AT was a single-looped macro-reentrant AT with a single LAAW gap as the critical area (Figure 1A and Figure 2, and Supplementary Video S1). Fourteen ATs with 14 conduction gaps were classified as this subtype, of which seven conduction gaps were located at the MVA end of LAAW line, four at the RSPV end of LAAW line, and three at the middle of LSPV-MVA line. The propagation of AT ran across the LAAW gap and around the MVA.

**Figure 3:**
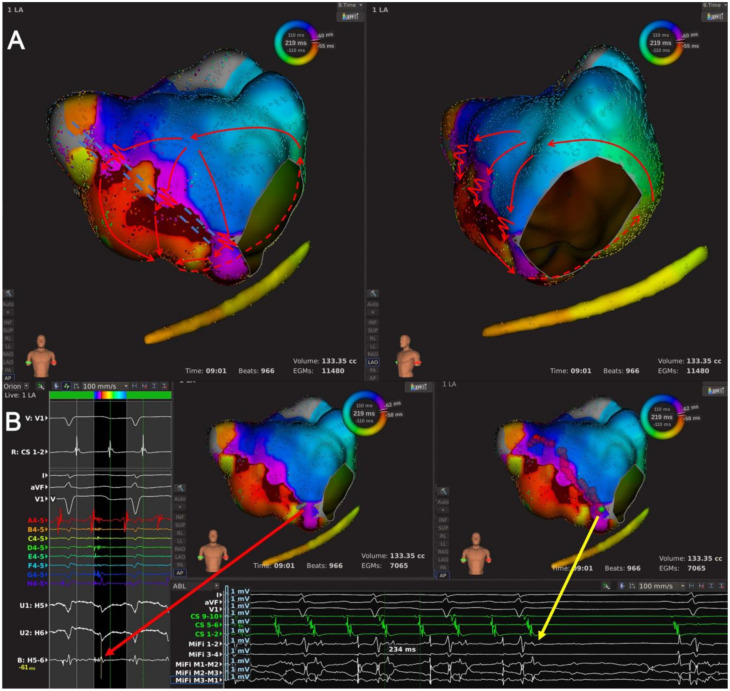
The high-density mapping of a Type 1.2 AT. (**A**) Activation map of a Type 1.2 AT from the anteroposterior view (left) and the left-anterior oblique view (right). The LA high-density mapping identified one LAAW block line from the RSPV to the MVA, with three conduction gaps at the MVA end, near the RSPV end, and in the middle of the LAAW line. The wavefront of AT ran down the LA septum, go along the MVA counterclockwise, and reached the LAAW after passing the MI, then the wavefront jumped across the LAAW conduction gaps almost simultaneously and returned to the LA septum. The red lines with arrows indicated the circuit of AT; the dashed red line indicated the circuit of AT behind the atrial structure; the jagged red lines indicated the wavefront passing the slow conduction areas; the double-dashed blue line indicated the LAAW block of line. (**B**) Local map potentials of the LAAW gap during AT (left) and at the moment of AT termination (bottom). The red dots indicated the ablation points; the green dot indicated the termination site; the arrowed red and yellow lines indicated the local potentials during AT and at the moment of AT termination, respectively.

Type 1.2: This subtype of AT featured pre-existing LAAW linear ablation with multiple conduction gaps (Figure 1B and Figure 3, and Supplementary Video S2). Three ATs with eight conduction gaps were classified as this subtype, and we identified three conduction gaps at the MVA end of the LAAW line, three at the RSPV end of the LAAW line, and two at the middle of the LSPV-MVA line. The wavefront ran around the MVA and went across all the LAAW gaps.

**Figure 4:**
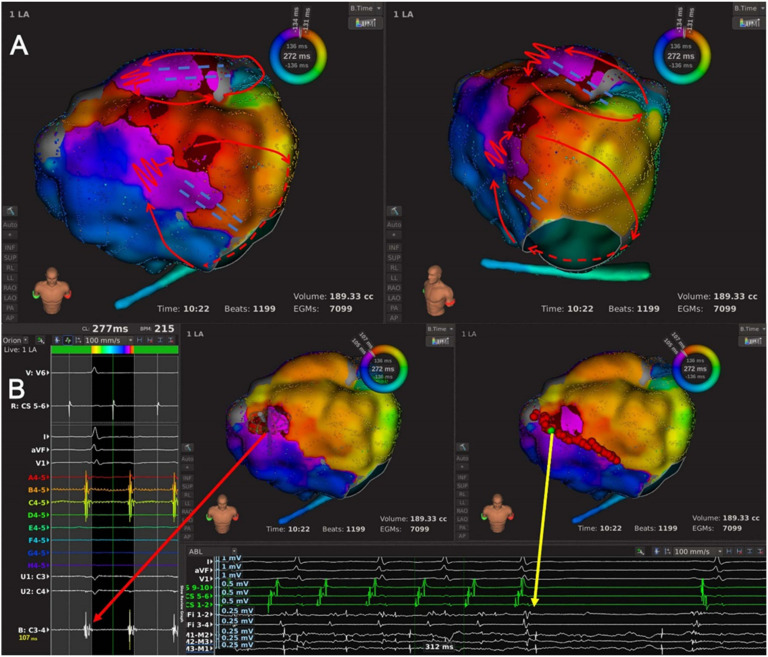
The high-density mapping of a Type 1.3 AT. (**A**) Activation map of a Type 1.3 AT from the anteroposterior view with additional cranial tilt (left) and the left-anterior oblique view with additional cranial tilt (right). The LA high-density mapping identified two block lines, including one LAAW block line from the RSPV to the MVA and one roof block line between the RSPV and the left superior pulmonary vein (LSPV). Two conduction gaps were identified, including one at the RSPV end of the LA roof line and the other at the RSPV end of the LAAW line. This was a dual-looped AT, and the activation propagated around the LA roof scar and the MVA simultaneously. The wavefront ran in LAAW from the right pulmonary vein antrum toward the LA free wall, turned around the MI and activated the LA posterior wall, and then divided into two branches: (1) the wavefront ran up and propagated along the LA roof line, went across the roof line conduction gap and returned to the LAAW; (2) the wavefront ran around the MVA, propagated upward in the LA septum, went across the LAAW conduction gap and returned to the LAAW. The red lines with arrows indicated the circuit of AT; the dashed red line indicated the circuit of AT behind the atrial structure; the jagged red lines indicated the wavefront passing the slow conduction area; the double-dashed blue lines indicated the LAAW and the roof block of lines. (**B**) Local map potentials of the LAAW gap during AT (left) and at the moment of AT termination (bottom). The red dots indicated the ablation points; the green dot indicated the termination site; the arrowed red and yellow lines indicated the local potentials during AT and at the moment of AT termination, respectively.

Type 1.3: This subtype of AT was dual-looped with LAAW conduction gap(s) as the critical area (Figure 1C and Figure 4, and Supplementary Video S3); it included two ATs. Two conduction gaps were identified, of which one was located in the middle of the LSPV-MVA line and the other between the septal and LAAW lesion line. The wavefront simultaneously propagated around the pre-existing LAAW scar and MVA.

Type 2: A macro-reentrant AT characterized by LA epicardial conduction connections, which is an indispensable part of the AT circuit. Since endocardial mapping could not measure epicardial connections accurately, only the locations of these epicardial connections were described. This type of AT included 11 ATs and can be further classified into two subtypes.

**Figure 5:**
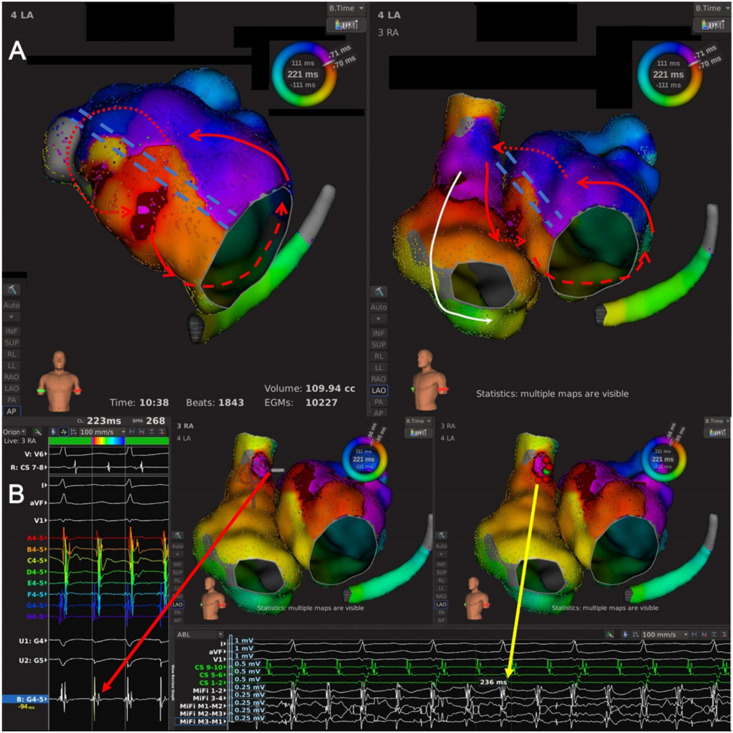
The high-density mapping of a Type 2.1 AT. (**A**) Activation map of a Type 2.1 AT from the anteroposterior view of LA (left) and the left-anterior oblique view of LA and RA (right). The high-density mapping identified one LAAW block line from the RSPV to the MVA, and the wavefront activated RA at the superior septum consistent with RA insertion of BB, ran down the septum and jumped to LA via the coronary sinus ostium. Then the wavefront ran around the MVA counterclockwise to LAAW and returned to RA via the insertion of BB near LA roof. The red lines with arrows indicated the circuit of AT; the dashed red line indicated the circuit of AT behind the atrial structure; the dotted red lines indicated the inter-atrial bypasses; the arrowed white line indicated passive activation; the double-dashed blue line indicated the LAAW block of line. (**B**) Local map potentials of the LAAW gap during AT (left) and at the moment of AT cycle length change (bottom). The red dots indicated the ablation points; the green dot indicated the ablation site of cycle length change; the arrowed red and yellow lines indicated the local potentials during AT and at the moment of AT cycle length change (from 221 ms to 236 ms), respectively.

Type 2.1: Single-looped bi-atrial reentrant ATs whose circuits used both LA and RA (Figure 1D and Figure 5, and Supplementary Video S4). This subtype included seven ATs, and an RSPV-MVA lesion line was identified in every AT map. The wavefront activated RA at the superior septum, consistent with RA insertion of BB, ran down the septum, and jumped to LA through fossa ovalis in five ATs and CS ostium in the other two ATs. Then, the propagation ran around MVA counterclockwise to LAAW and returned to LA insertion of BB to complete the circuit.

Among six patients with seven bi-atrial ATs (Type 2.1 AT), three patients (with three bi-atrial ATs) were after catheter ablation for AF, and the other three patients (50%, with four bi-atrial ATs) were after mitral surgery, of which the superior transeptal approach was performed in two (33.3%, with 3 bi-atrial ATs) and the transeptal approach was performed in the other.

**Figure 6:**
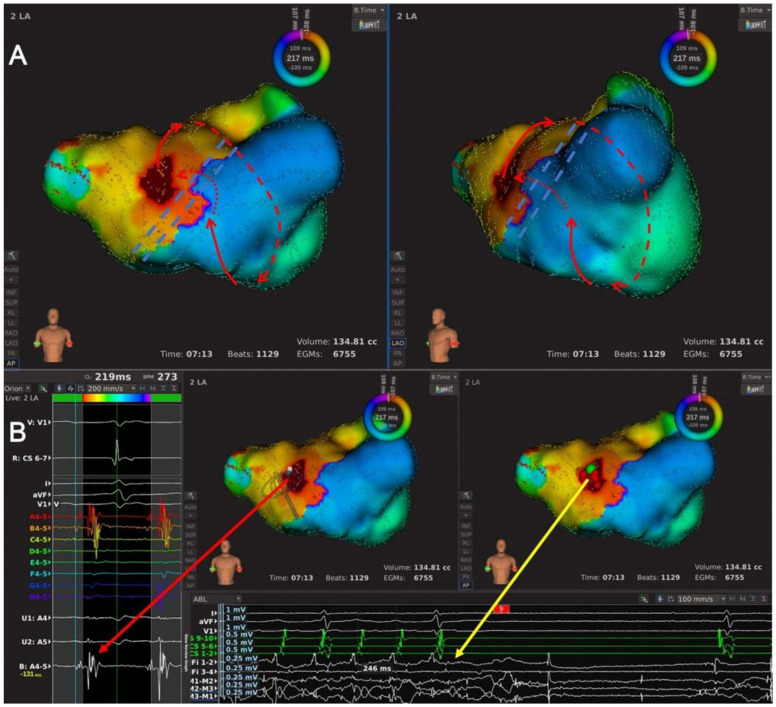
The high-density mapping of a Type 2.2 AT. (**A**) Activation map of a Type 2.2 AT from the anteroposterior view (left) and the left-anterior oblique view (right). The high-density mapping identified one LAAW block line stretching from LA septum extending to LA roof. The wavefront ran down the LA posterior wall, turned around the MI and activated LAAW, then jumped across the LAAW block line via the epicardial bypass and activate the other side in an areal pattern. Then the wavefront went upward around the LA roof and returned to the LA posterior wall. The red lines with arrows indicated the circuit of AT; the dashed red line indicated the epicardial bypass of the AT circuit; the dotted red line indicated the epicardial bypass; the double-dashed blue line indicated the LAAW block of line. (**B**) Local map potentials of the LAAW gap during AT (left) and at the moment of AT termination (bottom). The red dots indicated the ablation points; the green dot indicated the termination site; the arrowed red and yellow lines indicated the local potentials during AT and at the moment of AT termination, respectively.

Type 2.2 LAAW epicardial macro-reentrant AT. This subtype featured an epicardial connection crossing a pre-existing LSPV-MVA lesion line or a LA septal-roof lesion line (Figure 1E and Figure 6, and Supplementary Video S5). The wavefront began at the LAAW breakthrough above the lesion line, went around the LA roof and down along LA posterior wall, and then ran around LA lateral wall and across the lesion line via the epicardial connection. This subtype included four ATs.

**Figure 7:**
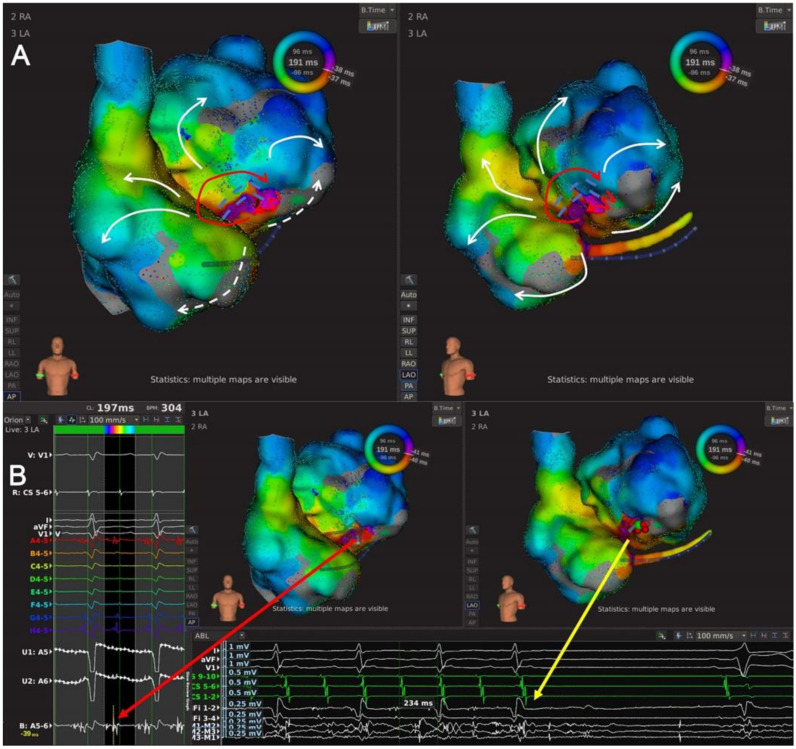
The high-density mapping of a Type 3 AT. (**A**) Activation map of a Type 3 AT from the anteroposterior view (left) and the left-anterior oblique view (right). The high-density mapping identified one minor LAAW block line near the MVA and one LAAW scar. The wavefront ran around the minor LAAW scar and propagated in a centrifugal pattern to activate other part of the LA. The red line with arrows indicated the circuit of AT; the jagged red line indicated the wavefront passing the slow conduction area; the white lines with arrows indicated passive activation; the white dashed lines indicated passive activation behind the atrial structure; the double-dashed blue line indicated a minor LAAW block of line. (**B**) Local map potentials of the LAAW gap during AT (left) and at the moment of AT termination (bottom). The red dots indicated the ablation points; the green dot indicated the termination site; the arrowed red and yellow lines indicated the local potentials during AT and at the moment of AT termination, respectively.

Type 3: LAAW micro-reentrant AT. This subtype included one AT, and the circuit ran around a minor LAAW block and propagated in a centrifugal pattern to activate the other part of LA (Figure 1F and Figure 7, and Supplementary Video S6).

### 3.5. Ablation Results and Follow Up

**Figure 8:**
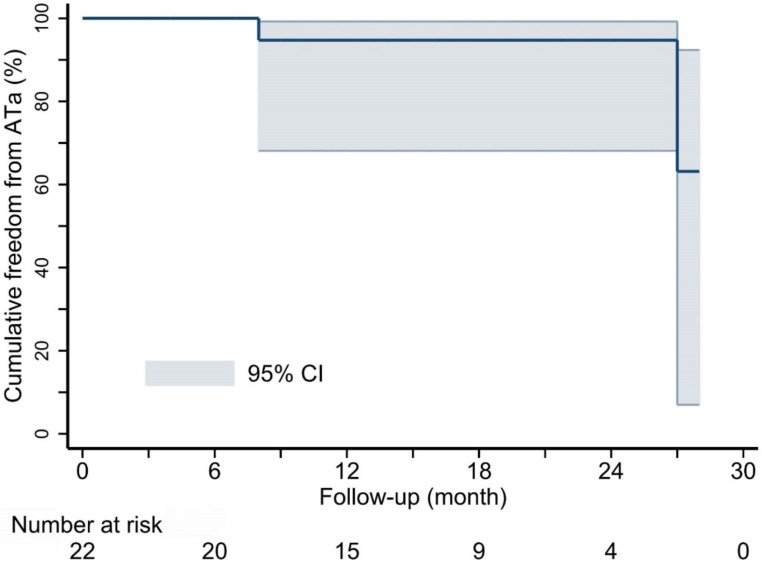
Kaplan-Meier survival curve showing cumulative freedom from atrial tachy-arrhythmia after ablation. ATa, atrial tachy-arrhythmia; CI, confidential interval.

Catheter ablation targeting conduction gaps on LAAW lines was performed in all 19 Type 1 ATs. After analyzing atrial activation and propagation maps, we further confirmed ablation targets by identifying residual potentials on LAAW line with conductive function. Effective ablation was obtained in all ATs, including termination in 12 ATs and circuit change in the other seven. Since the propagation ran around the MVA in these patients, mitral isthmus (MI) linear ablation connecting the MVA to the inferior margin of the left inferior pulmonary vein ostium was first performed in three ATs but was ineffective. These ATs were terminated by the following ablation targeting LAAW conduction gaps. Effective ablation was also obtained in seven Type 2.1 ATs, including termination in five ATs and circuit change in two. Meanwhile, in four Type 2.2 ATs, only one AT was terminated and the circuits changed in two ATs; the other patient restored sinus rhythm via cardioversion. All effective ablations for the 10 Type 2 ATs were achieved by targeting endocardial insertion of BB. The Type 3 AT was terminated by ablation targeting the two gaps in the LAAW line. At 16.0 ± 7.6 month follow-up, recurrent AT was observed in two patients (Figure 8).

## 4. Discussion

### 4.1. Main Findings

The present study reported the results of mapping and ablation of LAAW-related ATs after LAAW ablation or surgery via a high-density mapping system. The main findings were as follows. (1) Three types of LAAW scar-related ATs were identified in patients after iatrogenic LAAW intervention, i.e., mostly macro-reentrant AT (Type 1 and Type 2) and only rarely micro-reentrant AT (Type 3). (2) Epicardial conduction gaps were not uncommon in LAAW scar-related ATs, and ablation targeting the earliest insertion area was effective. (3) The application of a high-density mapping system could provide a precise AT circuit and help identify the ablation target in a time-efficient manner.

Although knowledge on LAAW-related AT remains limited, previous studies have made some efforts to explore its electrophysiological characteristics. Fukamizu et al. [10] reported ATs with a figure-eight circuit with loops around the mitral annulus, which is similar to Type 1.3 AT in our study. Wang et al. [11] reported peri-mitral AT as the most common macro-reentrant AT after mitral valve surgery. In our study, AT mechanisms in patients after cardiac surgery were more versatile, and only a third (4 out of 12) were single-looped peri-mitral ATs. Miyazaki et al. [12] and Zhu et al. [13] investigated LA septal/anterior wall reentrant AT, which is similar to Type 3 AT in our study. Although the LA scars were spontaneous and the study size was limited, the role of a low voltage area on LAAW in this subgroup should be further studied in the future. Rav-Acha et al. [14] reported a novel annotation technique in detail to improve accuracy in the detection of critical isthmus. In our study, the application of a new high-density mapping system allowed us to locate critical isthmus in a more efficient way with satisfactory accuracy for guiding following ablation.

### 4.2. Conduction Gaps and Reentrant AT

MI linear ablation is frequently used to treat peri-mitral AT; the LAAW line provides an alternative for the MI line, and could also be used in RFCA alone or combined with the MI line for non-paroxysmal AF to reduce the risk of recurrent AT. In the present study, conduction gap(s) on the LAAW line (Type 1 AT) was the major mechanism of LAAW scar-related AT(19/31) in 16 patients, of which most patients (10/16) were after RFCA for non-paroxysmal AF.

As revealed by our mapping results, the propagation ran around the MVA in all Type 1 ATs in patients after RFCA, indicating recovery of conduction in MI. Although MI linear ablation seemed to be an optional approach, the actual slow conduction areas revealed by high-density mapping were the conduction gaps on LAAW rather than MI. Moreover, compared with MI linear ablation, for which it is difficult to obtain a complete bi-directional block, ablation targeting LAAW gaps is easier to perform and is more reliable, and thus, should be taken as a preferable strategy. Furthermore, in all Type 1 ATs, the mechanisms were related to LAAW conduction gap(s), and the critical area was LAAW gaps in all Type 1 Ats. Therefore, it is reasonable that a complete block of the LAAW line is appropriate to prevent peri-mitral AT. Nonetheless, it is noteworthy that the slow conduction area, which is indispensable for reentry, was located on LAAW gap(s) rather than MI in all Type 1 ATs. This suggests that an incomplete block of the LAAW line may increase the risk of recurrent AT. Therefore, verification of the block of the LAAW line should be performed routinely after restoration of sinus rhythm. High-density mapping may be helpful for the identification of unobvious conduction gaps and/or slow conduction areas. LAAW gaps near the MVA were identified in 14 out of the 19 Type 1 ATs, possibly due to unstable catheter contact caused by the frequent and irregular movement of the MVA during AF ablation. Increased ablation energy near the MVA may be helpful to obtain solid ablation.

### 4.3. Epicardial Conduction Connection Related AT

Non-transmural iatrogenic intervention may produce epicardial conduction corridors which facilitate reentrant AT [15]. In the present study, epicardial conduction connection AT (Type 2) included bi-atrial AT (Type 2.1) and LAAW epicardial macro-reentrant AT (Type 2.2). One characteristic of Type 2 AT was that the location of the epicardial conduction connection was consistent with the distribution of BB and its branches in most Type 2 ATs (10/11), including seven Type 2.1 and three Type 2.2 ATs. This indicated that the BB and its branches may have served as corridors bypassing the LAAW linear lesion, facilitating epicardial macro-reentrant AT; hence, the risk of recurrent epicardial AT should be recognized in patients with LAAW iatrogenic intervention.

During ablation, a slight change in total CL of AT could be observed due to the change in the endocardial exit of epicardial AT. Subsequent ablation is needed to form an areal lesion to cover the area and obtain a complete block of the epicardial conduction corridor. Therefore, endocardial ablation for epicardial macro-reentrant LAAW AT can be complicated and requires close attention.

### 4.4. The Advantage of High-Density Mapping

High-density mapping provided a fast, detailed, and accurate way to investigate the mechanisms of AT and offered an intuitive perspective to guide subsequent ablation. It is even more valuable for the treatment of AT with complex mechanisms, as it shortens procedure times, limits ablative lesions, and lowers the risk of peri-procedural complications [16]. By taking large quantities of mapping points, the circuit and critical area of epicardial macro-reentrant AT and micro-entrant AT were also demonstrated clearly in our study. With a clear insight into the AT mechanism, unnecessary ablation lesions and subsequent iatrogenic scars can also be reduced, which may help lower the risk of recurrent scar-related atrial arrhythmia in long-term follow-up.

## 5. Limitations

The present study was limited by its retrospective design and relatively small sample size. In addition, entrainment mapping was not performed in all ATs (5/31); however, termination of AT and restoration of SR were obtained in such patients by ablation guided by a high-density mapping system. Furthermore, repeated mapping was not performed after the restoration of SR, and therefore, the data on the activation pattern of LA under SR remained unclear. Further studies are warranted to clarify the effect of completely blocking the LAAW line.

## 6. Conclusions

Three mechanisms of scar-related AT of LAAW were identified, two of which were related to LAAW conduction gaps. Epicardial AT or bi-atrial AT comprised a nonnegligible proportion in this subgroup of patients. A high-density mapping system could make it possible to determine the accurate mechanism of AT and serve as a guide following ablation.

## Figures and Tables

**Table 1:** Baseline characteristics of the patients.

Patient Number	Age	Gender	NYHA	Paroxysmal/ Persistent AT	Cardiac Diagnosis	LA Intervention	LAAW Intervention	Previous Ablation
1	62	0	2	Persistent	MR	MVR + TVP	surgical incision	0
2	83	1	2	Persistent	persistent AF	RFCA	linear ablation	1
3	78	0	2	Persistent	persistent AF	RFCA	linear ablation	1
4	73	1	2	Persistent	persistent AF	RFCA	linear ablation	1
5	71	1	2	Persistent	persistent AF	RFCA	linear ablation	1
6	67	1	2	Persistent	persistent AF	RFCA	linear ablation	1
7	74	0	3	Paroxysmal	MR	MVR	surgical incision	0
8	56	0	3	Persistent	MS	MVR	surgical incision	1
9	66	0	2	Persistent	MS	MVR	surgical incision	0
10	68	1	2	Persistent	persistent AF	RFCA	linear ablation	1
11	71	1	2	Paroxysmal	persistent AF	RFCA	linear ablation	1
12	68	0	2	Persistent	MS + AS	MVR + AVR	surgical incision	0
13	69	0	2	Persistent	persistent AF	RFCA	linear ablation	1
14	65	0	2	Persistent	MS	MVR + TVP	surgical incision	0
15	69	0	2	Persistent	persistent AF	RFCA	linear ablation	1
16	65	1	2	Paroxysmal	MS + MR + AR	MVR + AVR	surgical incision	0
17	64	0	2	Persistent	persistent AF	RFCA	linear ablation	1
18	52	0	1	Persistent	persistent AF	RFCA	linear ablation	1
19	83	0	2	Persistent	persistent AF	RFCA	linear ablation	1
20	58	0	1	Paroxysmal	persistent AF	RFCA	linear ablation	1
21	77	1	2	Persistent	MV prolapse	MVR + TVP	surgical incision	0
22	58	1	2	Persistent	MR + persistent AF	MVR + TVP + MAZE IV	surgical incision	1

NYHA, New York heart association; AT, atrial tachycardia; LA, left atrial; LAAW, left arial anterior wall; MR, mitral regurgitation; AF, atrial fibrillation; MS, mitral stenosis; MV, mitral valve; MVR, mitral valve replacement; TVP, tricuspid valve plasty; RFCA, radiofrequency catheter ablation; AVR, aortic valve replacement.

**Table 2:** The results of mapping and ablation of the patients.

Patient Number	AT Number	AT CL (ms)	RA Mapping Points	LA Mapping Points	Number of LAAW Gaps	AT Type	Effective Ablation Site	Effect of Ablation on AT
1	1	279	8151	9152	0	Type 2.1	Coronary sinus ostium	Termination
2	2	231	\	10,348	1	Type 1.1	LAAW gap near MVA	Circuit change
	3	254	\	9582	0	Type 2.2	Local breakthrough on LAAW	Circuit change
	4	370	\	10,164	0	Type 2.2	Local breakthrough on LAAW	None
3	5	223	7351	10,312	1	Type 1.1	LAAW gap near MVA	Termination
4	6	215	5645	12,537	2	Type 1.2	LAAW gaps (near MVA and RSPV)	Termination
5	7	235	\	13,642	1	Type 1.1	LAAW gap near MVA	Circuit change
	8	344	\	9824	1	Type 1.1	LSPV-MVA gap	Termination
6	9	191	\	11,465	1	Type 1.1	Local breakthrough on LAAW	Circuit change
7	10	203	\	8850	1	Type 1.1	LAAW gap near MVA	Circuit change
8	11	263	6931	15,098	1	Type 1.1	LAAW gap near MVA	Circuit change
	12	305	7326	13,972	0	Type 2.1	LA roof-LAAW junction corresponding to BB	Circuit change
	13	338	7493	13,275	0	Type 2.1	Fossa ovalis	Termination
9	14	488	4031	9180	0	Type 2.1	LA roof-LAAW junction corresponding to BB	Termination
10	15	228	\	9122	1	Type 1.1	LAAW gap near RSPV	Circuit change
	16	254	6478	9216	0	Type 2.1	LA roof-LAAW junction corresponding to BB	Termination
11	17	247	\	8588	1	Type 1.1	LAAW gap near MVA	Termination
12	18	265	6015	16,749	1	Type 1.3	LAAW gap on LA septum-roof line	Termination
13	19	219	\	7065	3	Type 1.2	LAAW gaps (near MVA, RSPV, and in the middle of LAAW line)	Circuit change
	20	235	\	8602	0	Type 2.1	Fossa ovalis	Termination
14	21	304	\	9931	1	Type 1.1	LSPV-MVA gap	Termination
15	22	255	\	13,188	1	Type 1.1	LAAW gap near RSPV	Termination
16	23	260	8219	9836	1	Type 1.3	LSPV-MVA gap	Circuit change
17	24	325	7291	10,227	0	Type 2.2	Local breakthrough on LAAW	Circuit change
18	25	221	5027	7089	0	Type 2.1	LA roof corresponding to BB	Circuit change
	26	236	6742	9674	1	Type 1.1	LAAW gap near RSPV	Termination
19	27	262	\	7099	1	Type 1.1	LAAW gap near RSPV	Termination
20	28	198	\	9155	2	Type 1.2	LAAW gaps (near MVA and RSPV)	Termination
21	29	217	9325	6757	0	Type 2.2	LA roof-LAAW junction corresponding to BB	Termination
22	30	223	\	11,703	1	Type 3	LAAW gap near MVA	Circuit change
	31	245	\	8329	1	Type 1.1	LSPV-MVA gap	Termination

AT, indicates atrial tachycardia; CL, cycle length; RA, right atrium; LA, left atrium; LAAW, left atrial anterior wall; MVA, mitral valve annulus; RSPV, right superior pulmonary vein; LSPV, left superior pulmonary vein; BB, the Bachmann’s bundle.

## Data Availability

Data can be acquired on reasonable request from the correspondence.

## Supplementary Materials

The following supporting information can be downloaded at: https://www.mdpi.com/article/10.3390/jcdd9080249/s1. Video S1, activation propagation of a Type 1.1 AT; Video S2, activation propagation of a Type 1.2 AT; Video S3, activation propagation of a Type 1.3 AT; Video S4, activation propagation of a Type 2.1 AT; Video S5, activation propagation of a Type 2.2 AT; Video S6, activation propagation of a Type 3 AT.
